# ACPA-positive and ACPA-negative rheumatoid arthritis differ in their requirements for combination DMARDs and corticosteroids: secondary analysis of a randomized controlled trial

**DOI:** 10.1186/ar4439

**Published:** 2014-01-16

**Authors:** Seth D Seegobin, Margaret HY Ma, Chanaka Dahanayake, Andrew P Cope, David L Scott, Cathryn M Lewis, Ian C Scott

**Affiliations:** 1 Department of Medical and Molecular Genetics, King’s College London, Guy’s Hospital, Great Maze Pond, 8th Floor Tower Wing, London SE1 9RT, UK; 2 Academic Department of Rheumatology, Centre for Molecular and Cellular Biology of Inflammation, 1st Floor, New Hunt’s House, Guy’s Campus, King’s College London, Great Maze Pond, London SE1 1UL, UK; 3 Department of Rheumatology, 3rd Floor, Weston Education Centre, King’s College Hospital, Cutcombe Road, London SE5 9RJ, UK

## Abstract

**Introduction:**

UK guidelines recommend that all early active rheumatoid arthritis (RA) patients are offered combination disease-modifying antirheumatic drugs (DMARDs) and short-term corticosteroids. Anti-citrullinated protein antibody (ACPA)-positive and ACPA-negative RA may differ in their treatment responses. We used data from a randomized controlled trial - the Combination Anti-Rheumatic Drugs in Early RA (CARDERA) trial - to examine whether responses to intensive combination treatments in early RA differ by ACPA status.

**Methods:**

The CARDERA trial randomized 467 early active RA patients to receive: (1) methotrexate, (2) methotrexate/ciclosporin, (3) methotrexate/prednisolone or (4) methotrexate/ciclosporin/prednisolone in a factorial-design. Patients were assessed every six months for two years. In this analysis we evaluated 431 patients with available ACPA status. To minimize multiple testing we used a mixed-effects repeated measures ANOVA model to test for an interaction between ACPA and treatment on mean changes from baseline for each outcome (Larsen, disease activity scores on a 28-joint count (DAS28), Health Assessment Questionnaire (HAQ), EuroQol, SF-36 physical component summary (PCS) and mental component summary (MCS) scores). When a significant interaction was present, mean changes in outcomes were compared by treatment group at each time point using t-tests stratified by ACPA status. Odds ratios (ORs) for the onset of new erosions with treatment were calculated stratified by ACPA.

**Results:**

ACPA status influenced the need for combination treatments to reduce radiological progression. ACPA-positive patients had significant reductions in Larsen score progression with all treatments. ACPA-positive patients receiving triple therapy had the greatest benefits: two-year mean Larsen score increases comprised 3.66 (95% confidence interval (CI) 2.27 to 5.05) with triple therapy and 9.58 (95% CI 6.76 to 12.39) with monotherapy; OR for new erosions with triple therapy versus monotherapy was 0.32 (95% CI 0.14 to 0.72; *P* = 0.003). ACPA-negative patients had minimal radiological progression irrespective of treatment. Corticosteroid’s impact on improving DAS28/PCS scores was confined to ACPA-positive RA.

**Conclusions:**

ACPA status influences the need for combination DMARDs and high-dose tapering corticosteroids in early RA. In CARDERA, combination therapy was only required to prevent radiological progression in ACPA-positive patients; corticosteroids only provided significant disease activity and physical health improvements in ACPA-positive disease. This suggests ACPA is an important biomarker for guiding treatment decisions in early RA.

**Trial registration:**

Current Controlled Trials ISRCTN32484878

## Introduction

Rheumatoid arthritis (RA) is a heterogeneous disease spanning several subsets. One crucial subdivision is defined by the presence or absence of anti-citrullinated protein antibodies (ACPA), termed ACPA-positive and ACPA-negative RA, respectively [1]. ACPA-positive RA has a worse prognosis with higher rates of erosive damage [2]. It also has different risk factors than ACPA-negative RA with most genetic associations [3,4] and environmental risks, such as smoking [5] and alcohol abstinence [6], predominantly linked to ACPA-positive disease. These disparities suggest that RA ACPA subsets might respond differently to treatment [7].

Current RA management focuses on early intensive therapies, often using combinations of disease-modifying antirheumatic drugs (DMARDs) and glucocorticoids with rapid escalation to biologics in refractory cases. Guideline recommendations for the treatment of early RA differ across countries. UK guidelines from the National Institute for Health and Care Excellence (NICE) advocate that all individuals with active RA are offered combination DMARDs with short-term glucocorticoids [8]. American College of Rheumatology (ACR) guidelines suggest reserving combination DMARDs for patients with markers of severe disease, such as ACPA positivity [9]. The European League Against Rheumatism (EULAR) guidelines also suggest a stratified treatment approach, advocating biologics in patients with poor prognostic markers like ACPA that are failing to attain remission or low disease activity with an initial treatment strategy of synthetic DMARDs [10]. There are, however, insufficient data on prognostic factors in randomized controlled trials (RCTs) of combination DMARDs and biologics to know which approach is best.

We used data from an RCT of combination DMARDs and corticosteroids in early RA - the Combination Anti-Rheumatic Drugs in Early RA (CARDERA) trial [11] - to examine whether responses to intensive combination treatments differ by ACPA status. Our primary aim was to examine if combination DMARDs and corticosteroids had different effects on radiological progression in ACPA-positive and ACPA-negative RA. Our secondary aims were to evaluate if any differential effects also extended to disease activity, disability and quality of life (QoL).

## Methods

### Ethical approval

The CARDERA trial was approved by the South Thames Multicentre Research Ethics Committee (REC Reference: MREC (1) 99/04). Further ethical approval was obtained to process the archived serum for ACPA status from the East of England - Essex Research Ethics Committee (REC Reference: 11/EE/0544). Informed consent was obtained from all patients recruited to the CARDERA trial.

### Subjects

The CARDERA trial recruited patients with early active RA (of less than two years duration) from 42 UK centers; its details have previously been reported [11,12]. Patients were randomized to one of four treatment arms: (1) monotherapy with methotrexate; (2) double therapy with methotrexate and ciclosporin; (3) double therapy with methotrexate and prednisolone; (4) triple therapy with methotrexate, ciclosporin and prednisolone. A factorial-design was adopted to allow the simultaneous evaluation of prednisolone and ciclosporin in a 2 × 2 design. Treatment groups were well matched with similar baseline characteristics [11]. Patients were assessed every 6 months (for 24 months). Missing data were imputed through last observations carried forward (undertaken in 19% of patients at 24 months). We restricted our current analysis to the 431 individuals (from 467 recruited) who had their sera archived at baseline and evaluable for ACPA.

### Serological assessments

ACPA-status was evaluated using the commercial ELISA assay, the Axis-Shield DIASTAT anti-CCP2 test (Axis-Shield, Dundee, UK). All samples were processed in duplicate with a cut-off of >5 units/ml taken as positive in keeping with the manufacturer’s instructions. Rheumatoid factor (RF) had been processed at recruiting center laboratories during the original trial.

### Study treatments

Study treatments comprised: (1) methotrexate (starting at 7.5 mg/week and increasing by 2.5 mg every two weeks to a final dose of 15 mg/week); (2) “step-down” prednisolone (based on the trial by Boers *et al. *[13], comprising 60 mg/day in week 1, tapering to 7.5 mg/day in weeks 7 to 28 and thereafter further reduced and stopped by week 36) started with methotrexate; (3) ciclosporin (based on the trial by Pasero *et al. *[14], comprising 1.5 mg/kg daily initially, increased gradually to a target dose of 3 mg/kg daily) started three months after methotrexate. Prednisolone and ciclosporin were given as active tablets or placebos. Intra-articular glucocorticoids (40 mg methylprednisolone with lignocaine) were given (on no more than six occasions) as required. Intramuscular glucocorticoids were allowed but only three doses of 120 mg of depot methylprednisolone could be given in a year.

### Outcomes

The following disease outcomes were assessed: (1) radiological damage - the onset of new erosions at 24 months and modified Larsen scores; (2) disease activity - disease activity scores on a 28-joint count (DAS28); (3) disability- Health Assessment Questionnaire (HAQ); (4) QoL- SF-36 physical (PCS) and mental (MCS) component summary scores and EuroQol.

### Statistical analysis

Baseline differences between ACPA-positive and ACPA-negative patients were evaluated using t-tests, Wilcoxon signed-rank tests or chi-squared tests depending on data type and distribution.

To establish whether treatment response differed by ACPA status, we used a two-staged approach; this minimized the potential for inflation of type I error associated with multiple testing. The first stage used a mixed-effects repeated measures ANOVA model to examine the effect of ACPA, treatment (coded categorically as one of the four randomized treatment arms) and time (assessment visit) on mean changes in each RA outcome (Larsen, DAS28, HAQ, EuroQol, PCS and MCS scores). The key component of this model was an ACPA*treatment interaction term, which established whether treatment responses differed by ACPA status.

**Figure 1:**
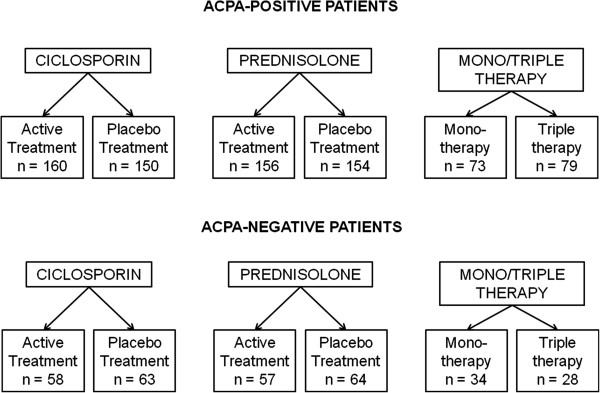
**Number of individuals in each treatment group stratified by ACPA status.** ACPA, anti-citrullinated protein antibody.

The second stage was restricted to outcomes associated with significant ACPA*treatment interactions and compared mean changes in these outcomes by treatment group at each time point using t-tests in ACPA-positive and ACPA-negative patients. Where the ratio between variances significantly differed from 1, Satterthwaite’s approximation was used to calculate the degrees of freedom for the critical t-statistic. This second stage allowed us to establish which treatments differed in their effects by ACPA status and how these differences changed over time. This analysis followed the original factorial grouping by comparing the following treatment groups (Figure 1): (a) active ciclosporin vs. placebo ciclosporin; (b) active prednisolone vs. placebo prednisolone; (c) triple therapy (methotrexate, cicosporin and prednisolone) vs. monotherapy (methotrexate).

In addition, we calculated odds ratios (ORs) for the development of new erosions with each treatment using binary logistic regression stratified by ACPA status.

*P*-values of <0.05 were considered significant. Analyses were performed using SAS version 9.3 (SAS Institute, Cary, NC, USA).

## Results

### Patient characteristics

Of the 431 RA cases, 310 (72%) were ACPA-positive and 121 (28%) were ACPA-negative. Baseline characteristics were similar between ACPA subsets with the exception of Larsen scores and RF status (Table 1). ACPA-positive patients had more radiological damage at baseline; the difference in median Larsen scores between ACPA-subsets was 3.00 units (*P* <0.001). Significantly more ACPA-positive patients were RF-positive (*P* <0.001). Both ACPA-positive and ACPA-negative patients had median ages in the fifth decade, were mainly female, had severely active RA (median DAS28 scores >5.1) of a short duration and moderate disability (median HAQ scores 1.62). QoL was moderately impaired (median EuroQol scores 0.58 to 0.60).

**Table 1:** Baseline characteristics by ACPA status

**Characteristic**	**ACPA-positive *(n = 310)***	**ACPA-negative *(n = 121)***	**Group difference**
Female (number; %)	208 (67%)	89 (74%)	*P* = 0.193^2^
RF positive (number; %)	244 (79%)	47 (39%)	*P* <0.001^2^
Age (years)	54.0 (46.0, 64.0)	55.0 (47.0, 62.0)	*P* = 0.661^1^
Disease duration (months)	2.00 (0.00, 5.00)	1.00 (0.00, 4.00)	*P* = 0.106^3^
Larsen score	7.50 (2.50, 21.25)*	4.50 (1.00, 9.50)	*P* <0.001^3^
DAS28	5.72 (4.91, 6.73)	5.96 (4.92, 6.85)	*P* = 0.305^1^
HAQ	1.62 (1.00, 2.12)	1.62 (1.12, 2.12)	*P* = 0.595^3^
EuroQol	0.60 (0.15, 0.68)	0.58 (0.08, 0.68)	*P* = 0.552^3^
SF-36 PCS	28.68 (23.24, 35.95)	28.70 (22.90, 35.56)	*P* = 0.713^3^
SF-36 MCS	38.64 (27.65, 53.71)	35.87 (25.37, 52.95)	*P* = 0.217^3^

All data are median interquartile range (IQR) unless otherwise stated; ^1^ = t-test; ^2^ = chi-squared test; ^3^ = Wilcoxon signed-rank test; *baseline Larsen scores missing in two ACPA-positive patients. ACPA, anti-citrullinated protein antibody; DAS28, disease activity scores on a 28-joint count; HAQ, Health Assessment Questionnaire; MCS, mental component summary; PCS, physical component summary; RF, rheumatoid factor.

### Radiological progression

The first analytical step, using the ANOVA model (Table 2), showed that treatment responses differed serologically with a significant ACPA*treatment interactive effect on changes in Larsen scores observed (*P* <0.001).

**Table 2:** ANOVA results for the effect of ACPA, treatment and time on changes in RA outcomes

	**Larsen**		**DAS28**		**HAQ**		**EuroQol**		**PCS**		**MCS**	
**Effects**	**F**	***P***	**F**	***P***	**F**	***P***	**F**	***P***	**F**	***P***	**F**	***P***
ACPA	31.90	<0.001	4.02	0.045	7.25	0.007	9.72	0.002	4.07	0.044	2.29	0.131
Time	16.83	<0.001	1.26	0.288	1.37	0.251	0.70	0.550	0.80	0.493	0.65	0.584
Treatment	9.93	<0.001	1.71	0.163	17.76	<0.001	11.47	<0.001	5.67	0.001	1.92	0.124
ACPA*Treatment	7.05	<0.001	3.99	0.008	0.48	0.696	2.94	0.032	3.22	0.022	1.84	0.138

F = F-statistic; *P = P*-value; ACPA*Treatment = ACPA*Treatment interaction term. ACPA, anti-citrullinated protein antibody; DAS28, disease activity scores on a 28-joint count; HAQ, Health Assessment Questionnaire; MCS, mental component summary; PCS, physical component summary; RA, rheumatoid arthritis.

**Figure 2:**
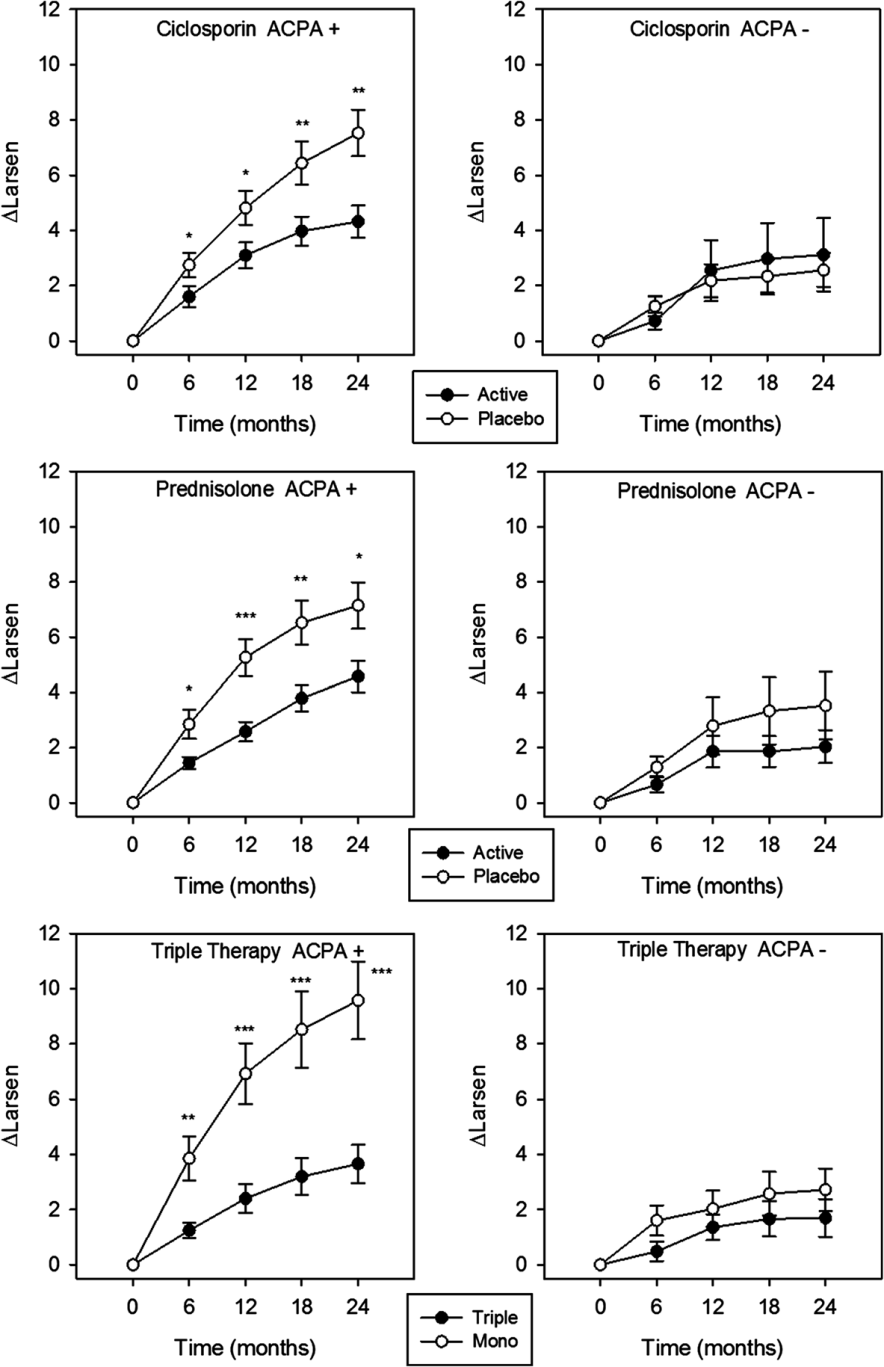
**Treatment effect on mean changes in Larsen scores in ACPA-positive and ACPA-negative patients.** Standard error bars are shown for each time point; *denotes significance at *P* <0.05; **denotes significance at *P* ≤0.01; ***denotes significance at *P* ≤0.001; no asterisk denotes *P* ≥0.05. ACPA, anti-citrullinated protein antibody.

The second analytical step, using the factorial approach, showed significant reductions in Larsen score progression in ACPA-positive patients receiving prednisolone, ciclosporin or triple therapy (Figure 2; Table 3). The magnitude of effect was similar with prednisolone and ciclosporin. Those receiving triple therapy had the largest reduction in radiological progression; mean Larsen score increases over 24 months were 3.66 (95% confidence interval (CI) 2.27 to 5.05) with triple therapy and 9.58 (95% CI 6.76 to 12.39) with monotherapy.

**Table 3:** Treatment effects on mean changes in Larsen and DAS28 scores in ACPA-positive and ACPA-negative RA

	**Time**	**Ciclosporin**			**Prednisolone**			**Triple vs. monotherapy**		
		**Ciclosporin**	**Placebo**	***P***	**Prednisolone**	**Placebo**	***P***	**Triple**	**Mono**	***P***
*Larsen*										
ACPA+	6	1.60 (0.37)	2.74 (0.44)	0.047	1.44 (0.22)	2.85 (0.53)	0.015	1.25 (0.28)	3.86 (0.80)	0.003
	12	3.09 (0.47)	4.81 (0.61)	0.027	2.58 (0.35)	5.26 (0.67)	0.001	2.41 (0.52)	6.92 (1.10)	<0.001
	18	3.97 (0.53)	6.43 (0.78)	0.010	3.79 (0.48)	6.52 (0.80)	0.004	3.20 (0.66)	8.52 (1.39)	0.001
	24	4.32 (0.58)	7.53 (0.84)	0.002	4.57 (0.57)	7.15 (0.84)	0.012	3.66 (0.70)	9.58 (1.41)	<0.001
ACPA-	6	0.72 (0.30)	1.25 (0.36)	0.270	0.67 (0.29)	1.30 (0.37)	0.182	0.48 (0.35)	1.60 (0.55)	0.093
	12	2.54 (1.11)	2.17 (0.59)	0.770	1.86 (0.58)	2.79 (1.04)	0.435	1.36 (0.47)	2.03 (0.66)	0.409
	18	2.97 (1.30)	2.33 (0.60)	0.660	1.86 (0.56)	3.33 (1.21)	0.275	1.66 (0.64)	2.57 (0.79)	0.388
	24	3.11 (1.32)	2.56 (0.61)	0.704	2.04 (0.60)	3.52 (1.22)	0.277	1.70 (0.69)	2.72 (0.77)	0.335
*DAS28*										
ACPA+	6	-1.61 (0.12)	-1.49 (0.13)	0.488	-1.97 (0.12)	-1.13 (0.11)	<0.001	-1.98 (0.18)	-0.99 (0.17)	<0.001
	12	-1.46 (0.12)	-1.19 (0.14)	0.147	-1.36 (0.13)	-1.29 (0.13)	0.716	-1.48 (0.18)	-1.14 (0.19)	0.190
	18	-1.49 (0.13)	-1.36 (0.14)	0.498	-1.50 (0.14)	-1.36 (0.13)	0.479	-1.64 (0.20)	-1.37 (0.20)	0.356
	24	-1.62 (0.13)	-1.38 (0.15)	0.211	-1.62 (0.14)	-1.38 (0.14)	0.203	-1.84 (0.19)	-1.36 (0.22)	0.087
ACPA-	6	-1.02 (0.21)	-1.42 (0.20)	0.173	-1.47 (0.24)	-1.00 (0.17)	0.111	-1.43 (0.33)	-1.32 (0.22)	0.792
	12	-0.94 (0.21)	-1.32 (0.21)	0.209	-1.01 (0.21)	-1.25 (0.21)	0.421	-0.73 (0.33)	-1.36 (0.33)	0.186
	18	-1.33 (0.24)	-1.52 (0.20)	0.552	-1.33 (0.23)	-1.51 (0.22)	0.579	-1.27 (0.37)	-1.62 (0.30)	0.462
	24	-1.16 (0.22)	-1.49 (0.20)	0.256	-1.32 (0.20)	-1.34 (0.21)	0.960	-1.27 (0.32)	-1.59 (0.30)	0.464

Data are mean changes (SE) unless otherwise stated; *P* = *P*-values from t-tests; Triple = triple DMARD therapy; Mono = monotherapy with methotrexate; time is in months. ACPA, anti-citrullinated protein antibody; DAS28, disease activity scores on a 28-joint count.

There were no significant treatment effects with any strategy in ACPA-negative patients. These individuals showed substantially less radiological progression (Figure 2; Table 3). The mean Larsen score increase in ACPA-negative patients treated with methotrexate monotherapy over 24 months was 2.72 (95% CI 1.15 to 4.29); for those receiving triple therapy the mean increase was 1.70 (95% CI 0.29 to 3.10).

Differences in radiological progression between ACPA-subsets were also seen in the proportion of patients developing new erosions (24% of ACPA-positive patients; 7% of ACPA-negative patients). Reductions in erosion development in ACPA-positive patients were similar with ciclosporin (OR 0.55; 95% CI 0.31 to 0.96; *P* = 0.032) and prednisolone (OR 0.56; 95% CI 0.32 to 0.99; *P* = 0.045) when compared with placebo. Triple therapy had the greatest impact on reducing new erosions when compared with monotherapy (OR 0.32; 95% CI 0.14 to 0.72; *P* = 0.003). Treatment had no significant impact on preventing erosions in ACPA-negative patients. The ORs for reduction in erosion development in ACPA-negative patients comprised 0.86 (95% CI 0.16 to 4.23; *P* = 1.00) and 0.89 (95% CI 0.17 to 4.38; *P* = 1.00) with ciclosporin and prednisolone, respectively, compared to placebo and 0.79 (95% CI 0.06 to 7.53; *P* = 1.00) for triple therapy compared with monotherapy.

### Disease activity

**Figure 3:**
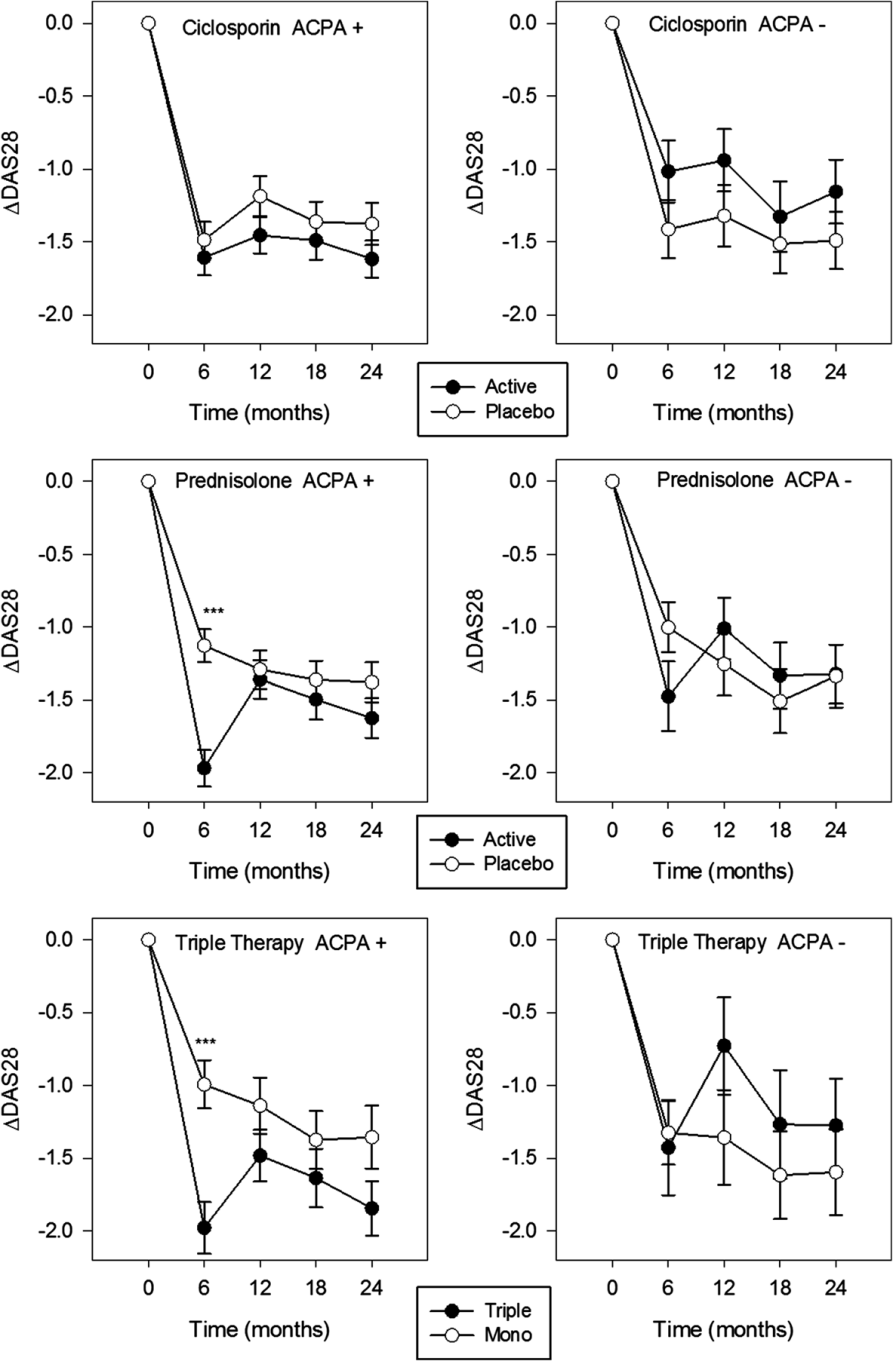
**Treatment effect on mean changes in DAS28 scores in ACPA-positive and ACPA-negative patients.** Standard error bars are shown for each time point; *denotes significance at *P* <0.05; **denotes significance at *P* ≤0.01; ***denotes significance at *P* ≤0.001; no asterisk denotes *P* ≥0.05. ACPA, anti-citrullinated protein antibody; DAS28, disease activity scores on a 28 joint count.

The ANOVA model (Table 2) showed a significant ACPA*treatment interactive effect on changes in DAS28 scores (*P* = 0.008). Subsequent factorial analysis by treatment showed that prednisolone (*P* <0.001) and triple therapy (*P* <0.001) significantly reduced DAS28 scores at six months in ACPA-positive patients (Figure 3; Table 3). No treatment effects were seen at subsequent time points. There were no significant treatment effects in ACPA-negative patients.

### Disability

The ANOVA model (Table 2) showed that although ACPA status (*P* = 0.007) and treatment (*P* <0.001) influenced changes in HAQ scores no ACPA*treatment interaction existed (*P* = 0.696). A factorial analysis was therefore not undertaken.

### Quality of life

#### *EuroQol*

**Figure 4:**
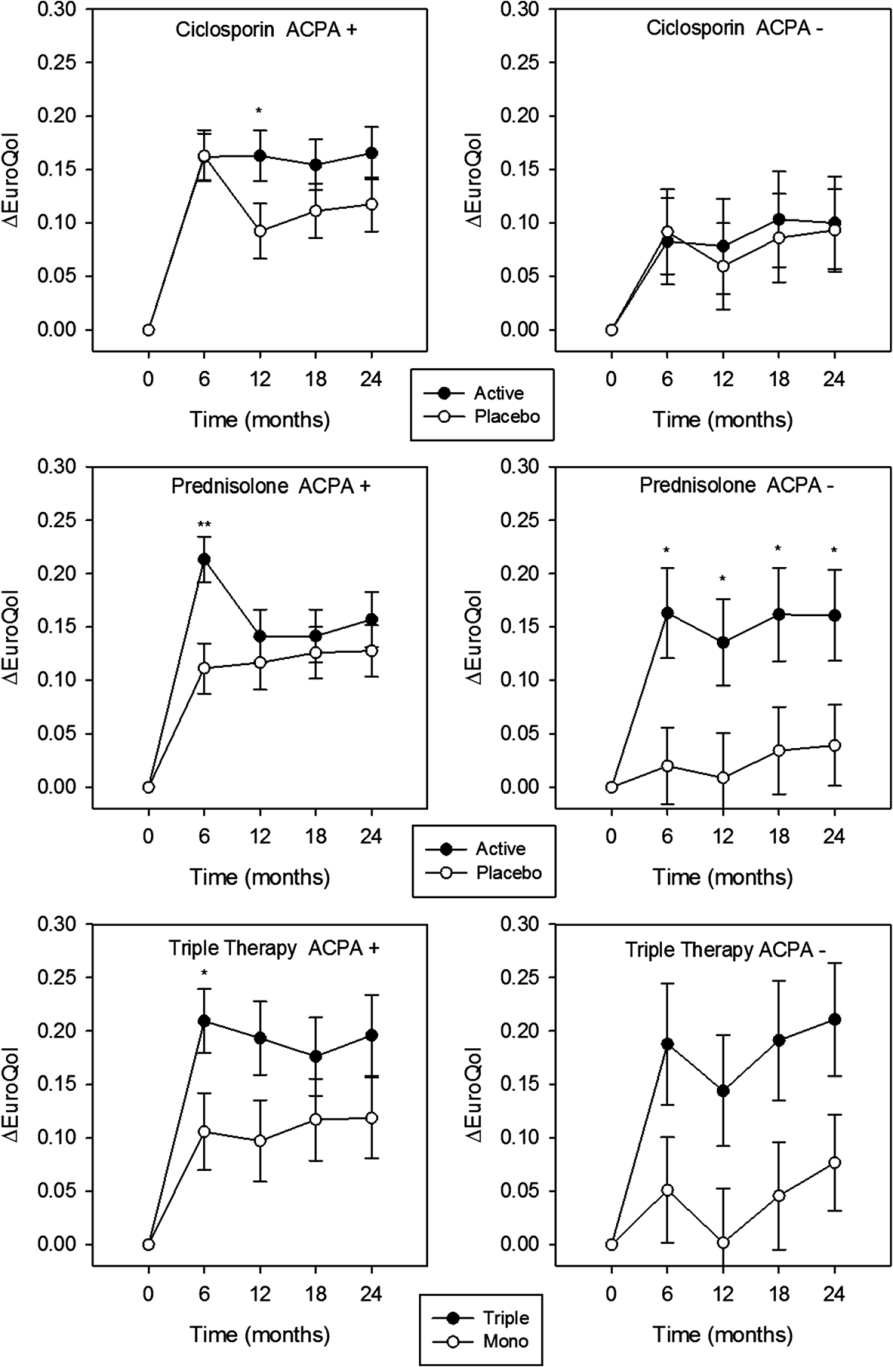
**Treatment effect on mean changes in EuroQol scores in ACPA-positive and ACPA-negative patients.** Standard error bars are shown for each time-point; *denotes significance at *P* <0.05; **denotes significance at *P* ≤0.01; ***denotes significance at *P* ≤0.001; no asterisk denotes *P* ≥0.05. ACPA, anti-citrullinated protein antibody.

The ANOVA model (Table 2) showed a significant ACPA*treatment interactive effect on changes in EuroQol scores (*P* = 0.032). Subsequent factorial analysis (Figure 4; Table 4) showed significant improvements in EuroQol scores at 6 months in ACPA-positive patients receiving prednisolone (*P* = 0.001) or triple therapy (*P* = 0.029); a significant effect was also seen at 12 months with ciclosporin (*P* = 0.044). A significant effect of prednisolone on EuroQol scores was seen at all time-points in ACPA-negative patients.

**Table 4:** Treatment effects on mean changes in EuroQol and PCS scores in ACPA-positive and ACPA-negative RA

		**Ciclosporin**			**Prednisolone**			**Triple vs. monotherapy**		
	**Time**	**Ciclosporin**	**Placebo**	***P***	**Prednisolone**	**Placebo**	***P***	**Triple**	**Mono**	***P***
*EuroQol*										
ACPA+	6	0.16 (0.02)	0.16 (0.02)	0.978	0.21 (0.02)	0.11 (0.02)	0.001	0.21 (0.03)	0.11 (0.04)	0.029
	12	0.16 (0.02)	0.09 (0.03)	0.044	0.14 (0.02)	0.12 (0.02)	0.481	0.19 (0.03)	0.10 (0.04)	0.063
	18	0.15 (0.02)	0.11 (0.03)	0.215	0.14 (0.03)	0.13 (0.02)	0.651	0.18 (0.04)	0.12 (0.04)	0.267
	24	0.17 (0.02)	0.12 (0.03)	0.176	0.16 (0.03)	0.13 (0.02)	0.403	0.20 (0.04)	0.12 (0.04)	0.152
ACPA-	6	0.08 (0.04)	0.09 (0.04)	0.876	0.16 (0.04)	0.02 (0.04)	0.011	0.19 (0.06)	0.05 (0.05)	0.074
	12	0.08 (0.04)	0.06 (0.04)	0.756	0.14 (0.04)	0.01 (0.04)	0.033	0.14 (0.05)	0.00 (0.05)	0.057
	18	0.10 (0.04)	0.09 (0.04)	0.776	0.16 (0.04)	0.03 (0.04)	0.036	0.19 (0.06)	0.05 (0.05)	0.058
	24	0.10 (0.04)	0.09 (0.04)	0.908	0.16 (0.04)	0.04 (0.04)	0.035	0.21 (0.05)	0.08 (0.05)	0.057
*PCS*										
ACPA+	6	7.96 (0.89)	5.15 (0.95)	0.031	9.15 (0.96)	4.01 (0.83)	<0.001	10.42 (1.30)	2.29 (1.16)	<0.001
	12	5.27 (0.84)	4.17 (0.94)	0.380	6.04 (0.93)	3.42 (0.84)	0.037	7.05 (1.23)	3.28 (1.27)	0.035
	18	6.11 (0.89)	3.87 (1.02)	0.097	5.51 (1.01)	4.53 (0.89)	0.472	7.77 (1.39)	4.58 (1.44)	0.114
	24	5.77 (0.96)	4.91 (1.03)	0.544	6.36 (1.03)	4.34 (0.95)	0.150	8.33 (1.46)	5.53 (1.49)	0.180
ACPA-	6	2.64 (1.41)	4.04 (1.21)	0.449	4.14 (1.31)	2.68 (1.29)	0.428	4.86 (2.11)	4.54 (1.78)	0.908
	12	3.27 (1.35)	4.93 (1.15)	0.349	3.70 (1.25)	4.52 (1.25)	0.646	3.25 (2.07)	5.61 (1.76)	0.386
	18	3.86 (1.38)	5.06 (1.24)	0.516	4.41 (1.40)	4.55 (1.23)	0.937	4.72 (2.11)	5.88 (1.67)	0.664
	24	4.65 (1.69)	5.17 (1.07)	0.793	5.51 (1.58)	4.40 (1.21)	0.575	7.44 (2.70)	6.48 (1.36)	0.753

Data are mean changes (SE) unless otherwise stated; *P* = *P*-values from t-tests; Triple = triple DMARD therapy; Mono = monotherapy with methotrexate; time is in months. ACPA, anti-citrullinated protein antibody; PCS, physical component summary.

#### *SF-36 PCS*

**Figure 5:**
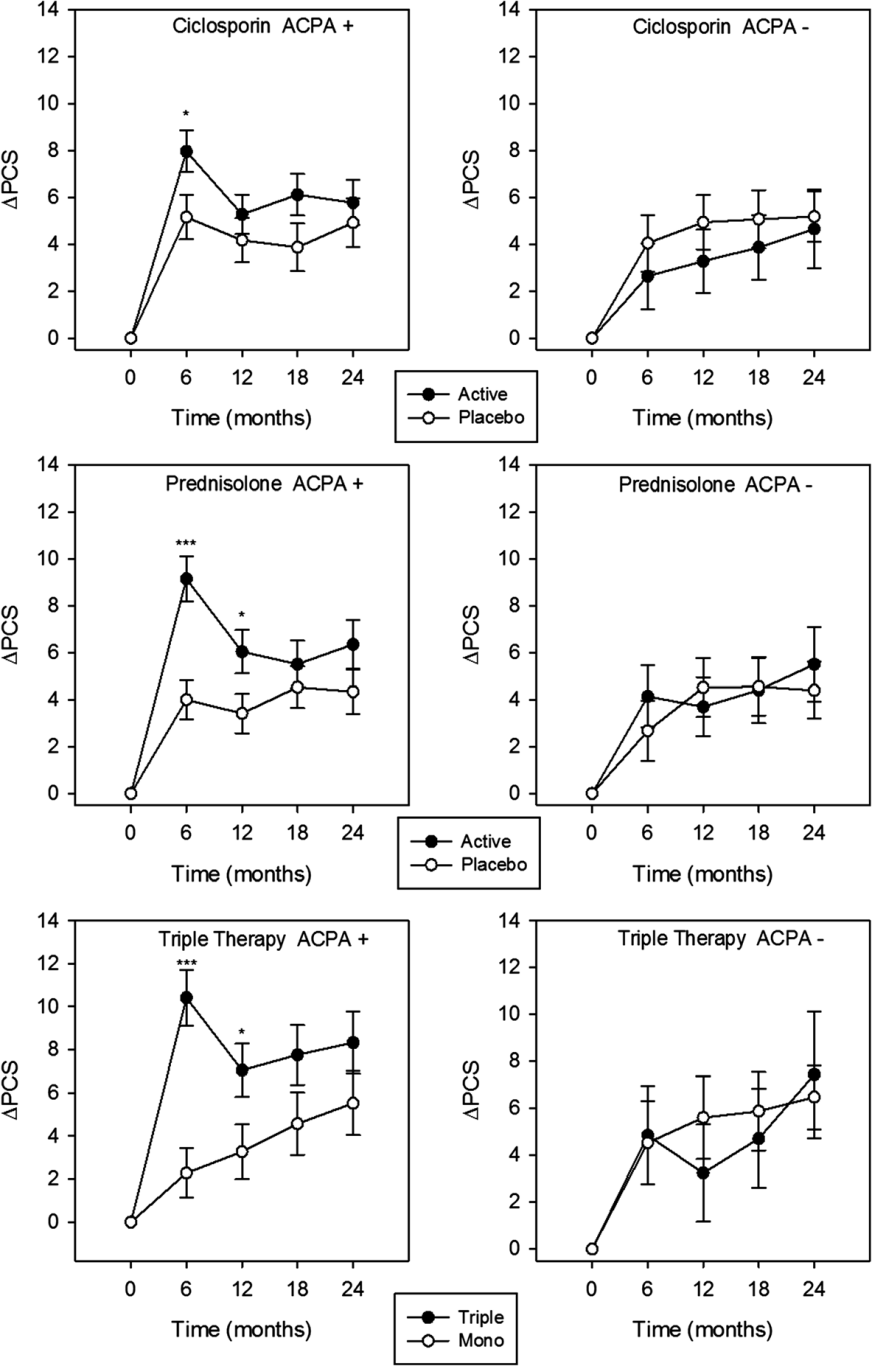
**Treatment effect on mean changes in PCS scores in ACPA-positive and ACPA-negative patients.** Standard error bars are shown for each time point; *denotes significance at *P* <0.05; **denotes significance at *P* ≤0.01; ***denotes significance at *P* ≤0.001; no asterisk denotes *P* ≥0.05. ACPA, anti-citrullinated protein antibody; PCS, physical component summary.

The ANOVA model (Table 2) showed a significant ACPA*treatment interactive effect on changes in PCS scores (*P* = 0.022). Factorial analysis (Figure 5; Table 4) showed that in ACPA-positive patients, prednisolone and triple therapy significantly improved PCS scores at 6 and 12 months; ciclosporin also improved PCS scores at 6 months (*P* = 0.031). In ACPA-negative patients no significant treatment effect on PCS scores was observed.

#### *SF-36 MCS*

The ANOVA model (Table 2) showed no significant associations between ACPA, time or treatment and MCS scores; no ACPA*treatment interaction was observed (*P* = 0.138). Factorial analysis was therefore not undertaken.

## Discussion

Our main finding is that combination DMARDs and high-dose tapering corticosteroids are only required to prevent radiological progression in patients with early active RA in whom ACPA is present. In ACPA-positive patients, methotrexate monotherapy resulted in considerable worsening of radiological damage; the average annual Larsen score increase was 4.8 units and 38% developed new erosions. This was significantly reduced with combination treatment; in ACPA-positive patients receiving triple therapy the average annual Larsen score increase was 1.8 units and 16% developed new erosions. In contrast, ACPA-negative patients had minimal radiological progression irrespective of the treatment strategy used; the average annual increases in Larsen scores were below the minimal clinically important difference (MCID) of 2.3 units [15] with all treatments and only 7% developed new erosions.

Our other finding was that the beneficial effect of high-dose corticosteroids on reducing disease activity and improving physical health was also confined to ACPA-positive RA. Only ACPA-positive patients had significant six-month improvements in DAS28 and PCS scores with double and triple therapy regimens incorporating prednisolone. Our findings are consistent with the IMPROVED study, which also found that high-dose corticosteroids had a significantly larger effect on improving disease activity and remission rates in ACPA-positive, as compared to ACPA-negative, inflammatory arthritis patients [16]. The mechanism underlying this differential steroid response is uncertain. The fact these improvements were not maintained over time in CARDERA is expected and consistent with the original COBRA study [13]. Our results support the use of high-dose tapering corticosteroids as a bridging therapy in early RA but suggest this treatment strategy would be best reserved for ACPA-positive patients.

The impact of ACPA status on EuroQol responses to combination DMARDs and corticosteroids was less clear, with similar EuroQol improvements observed in ACPA-positive and ACPA-negative patients receiving active prednisolone and triple therapy. Interestingly, ACPA-negative patients receiving placebo prednisolone or methotrexate monotherapy had substantially smaller EuroQol improvements (maximal increase of 0.04 and 0.08 units, respectively) when compared to ACPA-positive patients (maximal increase of 0.13 and 0.12 units, respectively). This suggests that methotrexate monotherapy could be more effective at improving QoL in ACPA-positive disease.

To our knowledge, research on the impact of ACPA status on responses to combination DMARDs and corticosteroids is limited to secondary analyses or extension studies of the BeST [17,18] and FIN-RACo [19] trials. Data from the BeST study support our finding that DMARD monotherapy is inadequate at preventing radiological progression in ACPA-positive RA; the presence of ACPA associated with radiological progression in individuals receiving monotherapy (OR for radiological progression: 12.6; 95% CI 3.0 to 51.9) but not combination therapy with DMARDs and corticosteroids (OR 1.7; 95% CI 0.5 to 5.4) [17]. This study also reported minimal radiological progression in ACPA-negative patients in all treatment groups. Although the FIN-RACo trial found that combination therapy reduced radiological progression in ACPA-negative, but not ACPA-positive patients, the study had a small sample size, allowed corticosteroids in both treatment arms and had no treatment restrictions for the last three years of follow-up [19]. The impact of ACPA-status on biologic responses has been studied in greater detail, particularly in national registries. Anti-tumor necrosis factor (anti-TNF) therapies appear more effective in ACPA-negative disease [20,21]. Conversely, T-cell and B-cell inhibition with abatacept and rituximab, respectively, appear more efficacious in ACPA-positive RA [22,23]. Taken together these findings suggest that treatment responses differ between ACPA-positive and ACPA-negative RA. This highlights a requirement for future RCTs of RA treatments to stratify their analyses by ACPA status.

Our results show that ACPA is an important prognostic biomarker in early RA, with its presence signaling a requirement for intensive combination treatment. The heterogeneous nature of RA alongside the increasing breadth of available therapies means that identifying predictors of treatment responses is a key research goal. Studies have identified several clinical parameters associated with good anti-TNF outcomes; these include not smoking, male gender and a younger age [20,24]. Genetic markers also offer promise with a recent large genome-wide association study reporting the first definitive genetic association (in the *CD84* gene) with anti-TNF response [25]. Other smaller studies suggest that stimulated whole blood cell pro-inflammatory cytokine levels [26] and serum proteins [27] may be useful in predicting anti-TNF efficacy. These findings are promising but lack clinical utility, since most markers require validation in larger cohorts or associate with only small differences in treatment response. Further work is required to identify predictors of treatment responses in RA.

Our study has a number of strengths. These include its large sample size, the involvement of multiple centers, the measurement of a wide range of outcomes and the use of two-year follow-up data. It also has several limitations. It was a secondary analysis of a published RCT and, therefore, neither its primary hypothesis nor its statistical analysis plan was pre-specified. ACPA status was unknown in 8% of patients, who were excluded from our analysis. One DMARD, ciclosporin, is not widely used in current practice. Fewer ACPA-negative patients were studied; however, the power to detect a MCID in Larsen scores between combination therapy and monotherapy treatment arms in ACPA-negative patients was higher (86%) than in ACPA-positive patients (55%). Finally, the maximal dose of methotrexate was 15 mg/week; higher doses are often used in contemporary clinical care [28].

Different guidelines, constructed using the same evidence base, have drawn alternative conclusions regarding the optimal treatment of early active RA. NICE guidelines recommend offering all patients combination DMARDs and short-term corticosteroids [8]. ACR and EULAR guidelines recommend adopting an individualized approach to treatment intensity based on prognostic factors, such as ACPA [9,10]. Our findings favor this latter approach. They show strong evidence that ACPA-positive patients benefit from intensive combination therapy but no evidence that combination treatments improve disease outcomes beyond methotrexate monotherapy in ACPA-negative patients. We recommend that future trials in early RA should consider ACPA status when evaluating treatment outcomes. When NICE and other clinical guidelines are updated, the heterogeneity of RA requires consideration, particularly the impact of ACPA-status on treatment requirements and responses.

## Conclusions

We have demonstrated that the requirement for, and response to, combination DMARDs and high-dose tapering corticosteroids differs between patients with ACPA-positive and ACPA-negative early RA. In our study, intensive combination therapy was only needed to prevent radiological progression in ACPA-positive patients. Additionally, corticosteroids only provided significant improvements in disease activity and physical health outcomes in ACPA-positive RA. These findings suggest that ACPA is an important biomarker for guiding treatment decisions in early RA. They support ACR and EULAR RA management guidelines, which recommend an individualized approach to treatment intensity based on prognostic factors such as ACPA.

## Abbreviations

ACPA: Anti-citrullinated protein antibodies; ACR: American College of Rheumatology; anti-TNF: Anti-tumor necrosis factor; CARDERA: Combination Anti-Rheumatic Drugs in Early RA; CI: Confidence interval; DAS28: Disease activity score on a 28-joint count; DMARD: Disease-modifying anti-rheumatic drug; EULAR: The European League Against Rheumatism; HAQ: Health assessment questionnaire; MCID: Minimal clinically important difference; MCS: Mental component summary; NICE: National Institute for Health and Care Excellence; OR: Odds ratio; PCS: Physical component summary; QoL: Quality of life; RA: Rheumatoid arthritis; RCT: Randomised controlled trial; RF: Rheumatoid factor.

## Competing interests

The authors declare that they have no competing interests.

## Authors’ contributions

ICS and DLS conceived and designed the study. ICS, MHYM and CD carried out the ACPA ELISA assays. SDS, ICS and CML performed the statistical analysis. SDS, ICS, CML and APC interpreted the data. ICS, SDS, CML, APC and DLS drafted the manuscript. All authors revised the manuscript critically for important intellectual content. All authors read and approved the final manuscript.
